# METTL3 promotes glycolysis and cholangiocarcinoma progression by mediating the m6A modification of AKR1B10

**DOI:** 10.1186/s12935-022-02809-2

**Published:** 2022-12-07

**Authors:** Jingli Cai, Zheng Cui, Jingyi Zhou, Bosen Zhang, Ruiqi Lu, Youcheng Ding, Hai Hu

**Affiliations:** 1 Center of Gallbladder Disease, Shanghai East Hospital, Institute of Gallstone Disease, Tongji University School of Medicine, No. 150, Jimo Road, Pudong New Area, Shanghai, China; 2 Department of Ultrasonic Medicine, Shanghai East Hospital, Institute of Gallstone Disease, Tongji University School of Medicine, Shanghai, China; 3 Department of Oncology, Shanghai General Hospital, Shanghai Jiao Tong University School of Medicine, Shanghai, China

**Keywords:** Cholangiocarcinoma, METTL3, AKR1B10, Glycolysis

## Abstract

**Objective:**

N6-methyladenosine (m6A) RNA methylation is involved in governing the mechanism of tumor progression. We aimed to excavate the biological role and mechanism of the m6A methyltransferase METTL3 in cholangiocarcinoma (CCA).

**Methods:**

METTL3 expression was determined by database and tissue microarray analyses. The role of METTL3 in CCA was explored by loss- and gain-of-function experiments. The m6A target of METTL3 was detected by RNA sequencing. The role of AKR1B10 in CCA was explored, and the association between METTL3 and AKR1B10 was confirmed by rescue experiments.

**Result:**

METTL3 expression was upregulated in CCA tissue, and higher METTL3 expression was implicated in poor prognoses in CCA patients. Overexpression of METTL3 facilitated proliferation, migration, invasion, glucose uptake, and lactate production in CCA cells, whereas knockdown of METTL3 had the opposite effects. We further found that METTL3 deficiency inhibited CCA tumor growth in vivo. RNA sequencing and MeRIP-qPCR confirmed that METTL3 enhanced AKR1B10 expression and m6A modification levels. Furthermore, METTL3 directly binds with AKR1B10 at an m6A modification site. A CCA tissue microarray showed that AKR1B10 expression was upregulated in CCA tissue and that silencing AKR1B10 suppressed the malignant phenotype mentioned above in CCA. Notably, knockdown of AKR1B10 rescued the tumor-promoting effects induced by METTL3 overexpression.

**Conclusion:**

Elevated METTL3 expression promotes tumor growth and glycolysis in CCA through m6A modification of AKR1B10, indicating that METTL3 is a potential target for blocking glycolysis for application in CCA therapy.

**Supplementary Information:**

The online version contains supplementary material available at 10.1186/s12935-022-02809-2.

## Introduction

Cholangiocarcinoma (CCA) describes a rare tumor originating from the bile duct epithelium that can involve the entire biliary tract [1]. According to the anatomical location, CAA can be classified into intrahepatic CCA and extrahepatic CCA. Approximately 60% of CCA cases occur in the perihilar region, 30% in the mid or distal bile ducts, and 6%-10% intrahepatically [2]. The incidence of this rare tumor in Western countries is low, with 5,000 new cases per year in the USA; however, the incidence of CCA in China is three times that in the USA [3]. Moreover, CCA is the most frequent invasive malignant tumor of the biliary tract, second only to hepatocellular carcinoma as the primary malignant tumor of the liver [4]. Due to silent clinical features, CCA patients usually progress to an advanced stage at the point of diagnosis, by which point surgical resection is challenging. Unfortunately, the effectiveness of other available systemic treatments is very limited, and the molecular mechanisms of CAA are still not fully understood for many reasons. Therefore, in-depth research on the molecular mechanism of CAA is urgently needed to provide a theoretical basis for the development of new and effective treatments.

N6-methyladenosine (m6A) is the most common methylation modification on mRNA molecules in eukaryotes. As an increasing number of m6A-related enzymes are recognized, such as methyltransferase-like 3 (METTL3), fat mass and obesity-associated gene (FTO), AlkB homolog 5 RNA demethylase (ALKBH5), and YTH domain family 1 (YTHDF1), the powerful biological functions of m6A modification have been gradually revealed. Increasing evidence suggests that m6A modification may contribute to carcinogenesis through different regulatory mechanisms, including the regulation of mRNA stability [5], localization and translation [6], transport [7], splicing [8], and RNA‒protein interactions [9]. Among these m6A-related enzymes, METTL3 was originally identified as a methyltransferase and is involved in tumor progression. For example, METTL3 synergizes with hepatitis B X-interacting protein (HBXIP) to regulate the abundance of m6A modification of hypoxia-inducible factor-1 alpha (HIF-1α), resulting in metabolic reprogramming and malignant progression of hepatocellular carcinoma cells [10]. METTL3 could also drive hepatocellular carcinoma progression by mediating m6A modification of ubiquitin-specific processing protease 7 (USP7) [11] or abnormal spindle-like microcephaly (ASPM) [12]. However, the role of METTL3 in CCA progression remains obscure.

Cancer cells are highly dependent on aerobic glycolysis for energy supply, known as the Warburg effect [13]. Aerobic glycolysis is defined by increased glucose uptake with preferential lactate generation, regardless of oxygen accessibility [14]. Aerobic glycolysis supports malignant tumor initiation and progression and is considered to be one of the primary characteristics of metabolic reprogramming in tumor cells [15]. Therefore, targeting the aerobic glycolysis pathway remains a promising therapeutic strategy for cancers. Moreover, several studies have shown that m6A-dependent glycolysis can prompt cancer progression. For example, METTL3 stabilizes hexokinase 2 (HK2) and solute carrier family 2-facilitated glucose transporter member 1 (SLC2A1) (also known as glucose transporter, GLUT1) by mediating m6A modification in an insulin-like growth factor 2 mRNA-binding protein (IGF2BP)2/3-dependent manner to activate the glycolysis pathway, resulting in the tumorigenesis of colorectal cancer [16]. METTL3 regulation is also involved in glycolysis metabolism in hepatocellular carcinoma [17], esophageal squamous cell carcinoma [18], and non-small cell lung cancer [19]. However, whether METTL3 mediates the m6A modification of glycolysis-related genes and participates in the progression of CCA deserves further study.

In the present study, we intended to reveal the biological role of m6A modification of aldo–keto reductase family 1 member B10 (AKR1B10) mediated by METTL3 in CCA progression. We clarified the expression patterns of METTL3 and AKR1B10 in CCA based on the results of database analysis and CCA tissue microarray and revealed the functions of METTL3 and AKR1B10 in CCA through in vitro and in vivo experiments. The regulatory roles of METTL3 and AKR1B10 in CCA were clarified by functional rescue experiments. Our study proposed that METTL3 may be a target of potential inhibitors for blocking glycolysis for application in CCA therapy.

## Materials and methods

### Processing of TCGA and GEPIA2 data

We characterized the expression profile of CCA RNA-seq datasets downloaded from TCGA-Cholangio carcinoma (CHOL) dataset and then the differential expression of eight m6A methylation-related genes (FTO, HNRNPA2B1, HNRNPC, METTL3, WTAP, YTHDC1, YTHDC2, and YTHDC2) between CCA and normal control samples were evaluated using R package and plotted into heatmap using R package.

The GEPIA2 database contained of 36 tumor tissues of CHOL and 9 adjacent tissues samples. We analyzed the differential expression of METTL3 and AKR1B10 between the CCA and adjacent tissues.

### Tissue microarray immunohistochemistry (IHC)

A CCA tissue microarray (No. LVC1202) was generated from 60 cancer tissues and paired pericarcinomas that purchased from Boster Biological Technology co.ltd. IHC staining for METTL3 and AKR1B10 were performed using the above microarray tissue blocks of CCA. Briefly, paraffin-embedded tissues were made into 6 μm sections following deparaffinization and hydration. Sections were repaired by high-pressure following incubated with 0.33% H_2_O_2_ in methanol to block endogenous peroxidases and incubated with 10% normal horse serum in TTBS to block non-specific binding. Next, sections were incubated with anti-METTL3 (1:100, Proteintech, 15073-I-AP) or anti-AKR1B10 (1:500, Abcam, ab192865) overnight at 4 °C and then incubated with horse anti-mouse biotinylated antibody. Finally, sections were with colored by chromogen of DAB and counterstained with hematoxylin. Pictures were captured and exported using NDP.view 2.0. IHC staining results of METTL3 were assigned 1–3 scores and AKR1B10 were assigned 0–3 scores based on staining intensity of positive cells and percentage of positive cells. The section with strong staining intensity and diffuse of positive cells was assigned 3 score; strong staining intensity and focal distribution of positive cells was assigned 2 score; weak- medium staining intensity of positive cells was assigned 1 score; no staining or non-specific staining was assigned 0 score. METTL3 scores of 1 and 2 were categorized as low expression group and 3 as high expression group. AKR1B10 scores of 0 and 1 were categorized as low expression group, scores of 2 and 3 as high expression group.

### Cell culture, lentivirus construction and transfection

Human liver bile duct carcinoma cell RBE and HCC9810 were purchased from Procell (China) and were maintained in RPMI 1640 with L-Glutamine (CORNING, China) containing 10% FBS (GIBCO, China) and 1% penicillin/ streptomycin (GIBCO, China) at 37 °C and 5% CO_2_.

To construct METTL3 stably overexpressed stable RBE cell line, the full-length of METTL3 was inserted into the lentiviral vector pLenti-EF1a-EGFP-P2A-Puro-CMV-3 × FLAG-WRPE (OE-METTL3 group) and then harvested-lentiviruses were infected with RBE cells using polybrene (hexadimethrine bromide, Sigma 107689-100MG). Blank lentiviral vector was served as negative control (Vector group).

To transient knockdown expression of METTL3 in HCC981 cells and AKR1B10 in RBE cells, we used small interfering RNA (siRNA) method. The synthesized sequence of siRNA targeted METTL3 (siMETTL3) or AKR1B10 (siAKR1B10) by GenePharma (Shanghai, China) were shown in Additional file 1: Table S1.

Besides, for stable knockdown of METTL3 expression used in animal study, lentiviruses vector pLKO.1 puro containing METTL3 shRNA (shMETTL3) and non-targeting scrambled shRNA (shNC) were purchased from GenePharma (Suzhou, China).

According to the manufacturer’s instructions, 5 μL of siMETTL3, siAKR1B10, shMETTL3 and shNC were diluted in 45 μL OPTI-MEM and then mixed with 10 μL Lipofectamine 2000 reagent pre-diluted with 45 μL OPTI-MEM. The mixture was added into cells and cultured for 24 h before further efficiency verification experiments.

### RT-qPCR analysis

TRIzol (Invitrogen Life Technologies) was applied for isolating total RNA from RBE cells and HCCC-9810 cells. Quality qualified RNA reverse-transcription into cDNA was carried out using High Capacity cDNA Reverse Transcription kit (Applied Biosystems) and then mRNA expression of METTL3, AKR1B10, and GAPDH were measured by QuantStudio 6 Flex Real-Time PCR System (Thermo Fisher Scientific) with FastStart Universal SYBR Green Master mix (Takara, China) according to the product’s protocol. Relative expressions of genes were normalized to GAPDH using 2^−ΔΔCq^ method. The primers were listed in Additional file 1: Table S1.

### Western blot

Total protein concentration was measured by the BCA protein assay kit (Thermo scientific, USA). Then, 20 μg proteins were resolved by 10% SDS-PAGE and transferred onto PVDF membranes following blocking for nonspecific binding with 5% nonfat milk at 25 °C for 2 h. The membranes were incubated with anti-METTL3 (1:2000, Proteintech, 15,073-I-AP), anti-AKR1B10 (1:1000, Abcam, ab192865), and anti-GAPDH (1:1000, Proteint, 60004-1-Lg) at 4 °C overnight. After that, membranes were incubated with Goat Anti-Mouse IgG H&L (HRP) (1: 10,000, Abcam, ab205719) at 25 °C for 1 h. Immunore-activity was imaged by Bio-Rad ChemiDoc XRS system and quantified by Image J.

### CCK8 assays

One hundred microliter cells with a density of 1 × 10^5^ cells/well were seeded in 96-well plates and cultured for 24 h. Then, at each indicated times (0 h, 24 h, 48 h, 72 h, 96 h), 10 μL CCK-8 solution (Dojindo, Japan) was added and was incubated for another 1 h at 37 °C. The optical density was read at 450 nm using a Multiskan FC microplate reader (Thermo Fisher Scientific).

### Cell apoptosis detection using TUNEL

Cell apoptosis was detected by TUNLE assay using a One Step TUNEL apoptosis kit (red Tunnelyte™ CY3 fluorescence detection) (C1089, Beyotime, China) according to the product instruction. Briefly, adherent CCA cells were washed with PBS and then fixed in immunostaining fixative solution (P0098, Beyotime, China) followed by permeabilized in immunostaining strong permeable solution (P0097, Beyotime, China). After that, cells were incubated with TUNEL solution for 1 h and sealed with anti-fluorescence quenching sealing tablets. Lastly, cells were photographed on a fluorescence microscope.

### Transwell assays

Cell migration and invasion were accessed by using a Transwell assay. The Transwell chamber was coated with 0.8 μm Matrigel (354480, BioCoat) for cell invasion assay, otherwise for migration assay. CAA cells were seeded into the upper chamber containing serum-free medium and complete medium was added to the lower chamber as a chemoattractant. The cells were cultured 24 h at 37 °C. The migrated cells to the lower chamber was photographed and calculated in three randomly fields under an inverted light microscope. The invaded-cells arriving at the lower chamber were fixed in 10% formaldehyde for 15 min and stained with 0.1% crystal violet for 10 min, and finally photographed in three randomly fields.

### Glucose uptake and lactate production assay

Relative glucose uptake required by tumor cells was measured by Glucose Uptake Fluorometric Assay Kit (MAK084, Sigma-Aldrich, USA), and relative lactate production was measured by Lactic Acid Content Assay Kit (D799851-0050, Sangon, China) according to the technical bulletin provided by manufacturer.

### Subcutaneous xenograft tumor model

A total of twelve 4-week-old female BALB/c nude mice were purchased from Shanghai SLAC Laboratory Animal Company and were randomly divided into two groups: ShMETTL3 group (n = 6) and Vector group (n = 6). Mice were single housed at room temperature (21–26 °C) on a nature light cycle for one week before experiments to adapt laboratory environment. All mice were provided free access to diet and water. HCCC-9810 cells with METTL3 knockdown were digested by trypsin and made into single cell suspension. Next, 2 × 10^6^ HCCC-9810 cells were subcutaneously injected into axillary of mice. After injection, mice were continued to be raised normally for 3 weeks. Tumor volume (length × width × width × 0.5) was measured every 3 days using caliper. Mice were euthanized using CO_2_ inhalation after the last measurement, tumor was collected and weighted.

### Transcriptome sequencing

RNA sequencing was performed at Yingbio Technology (Shanghai, China) using an Illumina HiSeq 2500. For differentially expressed genes (DEGs) identification, the thresholds was Log2fold change (FC) > 1 or < − 1 and false discovery rate (FDR) < 0.05. Gene onology (GO) and Kyoto Encyclopedia of Genes and Genomes (KEGG) were carried out for the DEGs using R package. Transcriptome sequencing was repeated in three replicates.

### RNA immunoprecipitation (RIP) and m6A RIP qPCR (MeRIP-qPCR)

Total RNA was extracted from RBE cells-overexpressed METTL3 or not, and then isolated mRNA was purified by Dynabeads mRNA Purification Kit (Invitrogen, USA) according to the manufacturer’s instructions. Next, purified mRNA was fragmented by RNA Fragmentation Reagent (Invitrogen, USA) before immunoprecipitation. After that, the anti-m6A antibodies or anti-METTL3 were conjugated to protein magnetic beads for immunoprecipitation, anti-immunoglobulin G (IgG) was served as negative control. Finally, RNA was eluted from RNA–protein immunocomplexes followed by RT-qPCR analysis.

### Actinomycin D assay

RBE cells overexpressed METTL3 or NC were seeded in a 12-well plate and cultured for 24 h. Then, 5 μg/mL actinomycin D were added into cells and cultured another 0 h, 3 h, 6 h, 9 h, and 12 h following cell collection. Once collection, the total RNA was extracted from these RBE cells used for RT-qPCR as described above.

### Dual-luciferase reporter assays

For m6A reporter assays, the wild-type of AKR1B10 sequence and the mutated at m6A motif 1 (mut-1, m6A was replaced by G), at m6A motif 2 (mut-2, m6A was replaced by G), and at m6A motif 3 (mut-3, m6A was replaced by C) were inserted into XhoI/NotI site of the psiCHECK-2 luciferase reporter vector. Then, these recombinant plasmids were transfected into RBE cells overexpressed METTL3 and NC using Lipofectamine 2000 reagent as described above.

### Statistical analysis

Data analysis was performed by GraphPad Prism 9.0 and data were presented as mean ± SD. Kolmogorov–Smirnov test was used for evaluating the data normality, and Levene test was used for evaluating homogeneity of the variance of data. One-way ANOVA with Tukey test for three groups and t test for two groups were utilized when the data was normality and homogeneous. The chi-square test was used to evaluate the correlation between molecular expression and clinical data. P value less than 0.05 was considered significant.

## Results

### METTL3 expression is upregulated in CCA tissues

**Fig. 1:**
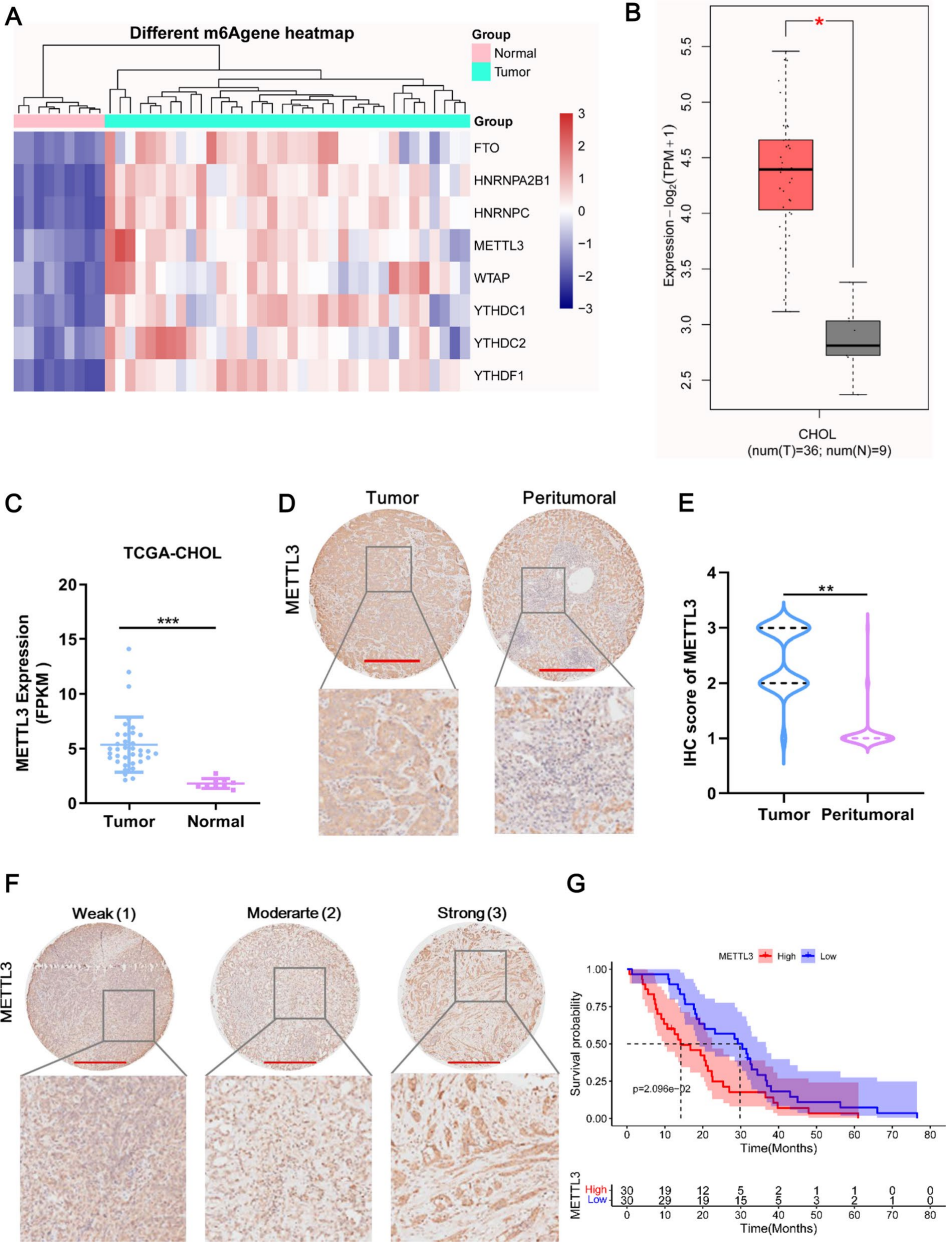
METTL3 expression is upregulated in CCA tissues. **A** Heatmap of m6A methylation-related differentially expressed mRNAs between CCA tissues and normal tissues. These differentially expressed mRNAs were identified by using TCGA-CHOL data. Red and blue indicate upregulated and downregulated mRNAs in CCA tissue compared with normal tissue, respectively. **B** METTL3 expression in the GEPIA2 dataset. The red box indicates CCA tumor tissue, and the gray box indicates normal tissue. **C** METTL3 expression in the TCGA-CHOL dataset. Tumor tissue, n = 36, normal tissue, n = 9. **D** Representative image of METTL3 expression in tumor and peritumoral tissue in the CCA tissue microarray. Compared with peritumoral tissues, METTL3 was overexpressed in CCA tissues, as revealed by IHC staining for METTL3 in a CCA tissue microarray (n = 60). Scale bar: 500 μm. **E** The METTL3 score in CCA tissues was significantly higher than that in peritumoral tissues. The sample sizes were 60 for the CCA tumor and peritumoral tissues. **F** Representative image of METTL3 expression at three levels in the CCA tissue microarray. METTL3 expression was scored at three levels (weak, moderate, and strong) on the basis of IHC staining intensity. Scale bar: 500 μm. **G** The overall survival analysis showed that high expression of METTL3 (red line) indicated a poor prognosis in patients with CCA. The median was used to define the cutoff, which was less than 3 for the low group. *Means P < 0.05, **means P < 0.01, ***means P < 0.001

To obtain m6A methylation-related genes in CCA, we analyzed TCGA-CHOL data. A total of 8 m6A methylation-related genes were differentially expressed between CCA and normal tissues (Fig. 1A). Among these 8 genes, METTL3 showed the highest fold-change of upregulation in CCA (Fig. 1C). The GEPIA2 dataset supported that METTL3 was significantly accumulated in CCA tissue compared with normal tissues (Fig. 1B). Specifically, TIMER analysis (http://timer.comp-genomics.org/) showed that METTL3 was upregulated in a variety of tumors, including CHOL (Additional file 2: Fig. S1A). Accordingly, we focused primarily on METTL3. To determine the expression of METTL3 in CCA tissues, we performed IHC staining for METTL3 in a CCA tissue microarray study that enrolled 60 patients. Compared with peritumoral tissues, METTL3 was overexpressed in CCA tissues (Fig. 1D). METTL3 expression was scored at three levels on the basis of IHC staining intensity, and the METTL3 score in CCA tissues was significantly higher than that in peritumoral tissues (Fig. 1E, F). According to the METTL3 score, we found that METTL3 expression was significantly correlated with TNM stage but not with the other clinicopathological characteristics (Table 1). The survival analysis showed that high expression of METTL3 indicated a poor prognosis in patients with CCA (Fig. 1G). Collectively, METTL3 expression is upregulated in CCA and is associated with CCA progression.

**Table 1:** Relationship between METTL3 and AKR1B10 expression and the clinicopathological characteristics in CCA tissue microarray

Tumor	METTL3 (n = 60)		χ^2^	P value	AKR1B10 (n = 44)		χ^2^	P value
	Low	High			Low	High		
Age			0.06787	0.7945			1.245	0.2645
< 60	16	18			15	13		
≥ 60	14	12			5	11		
Gender			0.2820	0.5954			2.146	0.1430
Female	10	13			12	8		
Male	20	17			8	16		
TNM stage			4.565	0.03263			1.426	0.2324
T1–T2	27	19			18	17		
T3–T4	3	11			2	7		
Metastasis			1.404	0.2361			0.3536	0.5521
Yes	0	3			1	1		
No	30	27			19	23		
Relapse			0.1042	0.7469			0.4596	0.4978
Yes	24	24			15	21		
No	6	6			5	3		
Greatest tumor diameter (cm)			0.06944	0.7921			0.4016	0.5263
< 5	11	13			8	13		
≥ 5	19	17			12	11		
Tumor-free survival (month)			0.6027	0.4376			0.1291	0.7194
< 4	12	16			12	12		
≥ 4	18	14			8	12		

### METTL3 promotes glycolysis and the malignant phenotype of CCA cells in vitro and in vivo

**Fig. 2:**
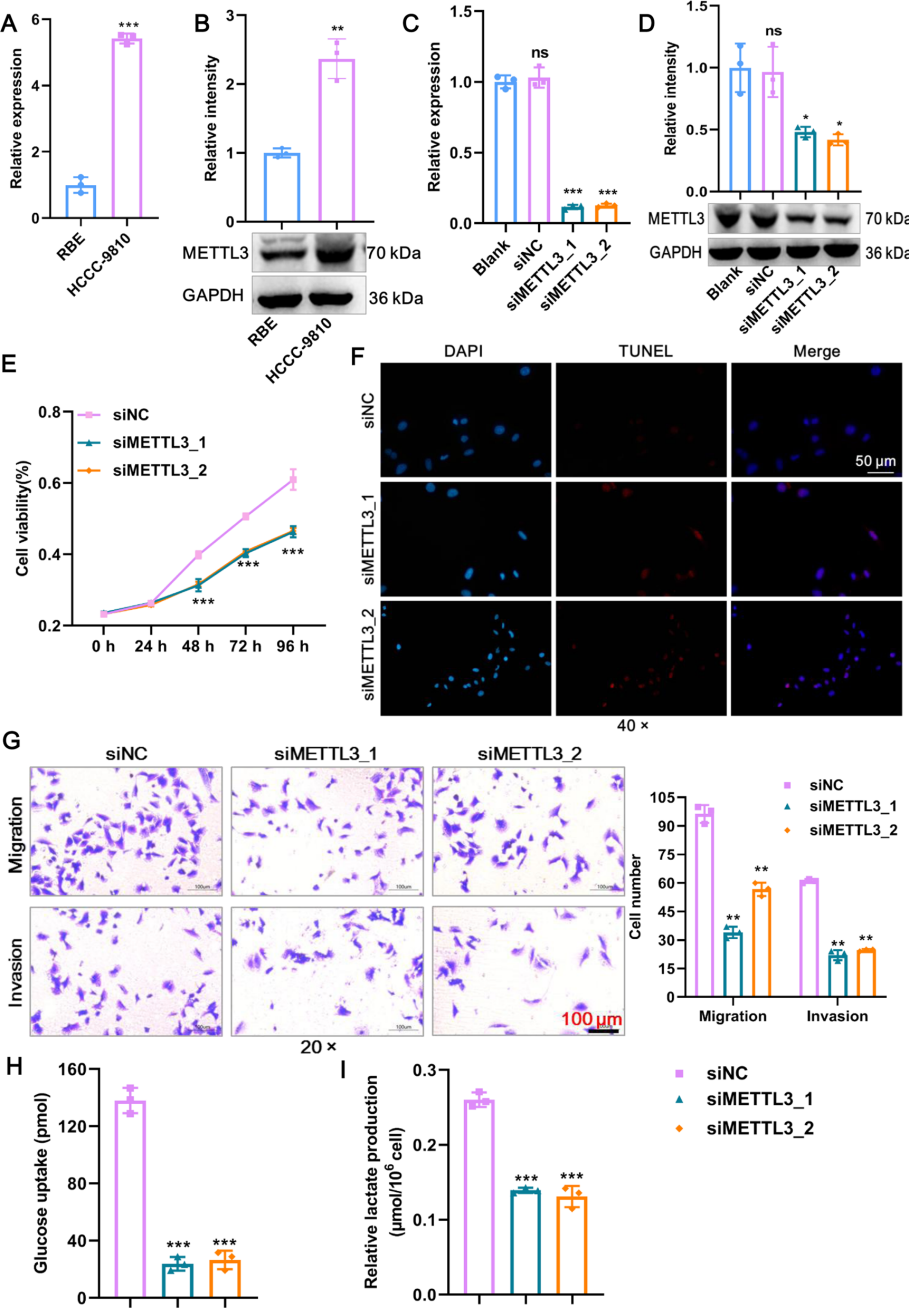
METTL3 promotes glycolysis and the malignant phenotype of CCA cells in vitro*. A* METTL3 mRNA expression in HCCC-9810 cells and RBE cells was detected by RT‒qPCR. **B** METTL3 protein expression in HCCC-9810 cells and RBE cells was detected by western blot. **C** The knockdown efficacy of two siRNAs against METTL3 mRNA expression in HCCC-9810 cells was confirmed by RT‒qPCR. **D** The knockdown efficacy of two siRNAs against METTL3 protein expression in HCCC-9810 cells was confirmed by western blot. **E** The proliferation of HCCC-9810 cells after knockdown of METTL3 was detected by CCK8. **F** TUNEL staining was used to detect HCCC-9810 cell apoptosis after METTL3 knockdown. Scale bar: 50 μm. **G** The migration and invasion of HCCC-9810 cells after METTL3 knockdown were detected by Transwell assays. The numbers of migrating and invading cells were counted for comparison. Scale bar: 100 μm. **H** Glucose uptake of HCCC-9810 cells after knockdown of METTL3 was detected using a commercial kit. **I** Lactate production in HCCC-9810 cells after knockdown of METTL3. **Means P < 0.01, ***means P < 0.001

To evaluate the role of METTL3 in CCA, we first detected endogenous mRNA expression of the METTL3 gene in two CCA cell lines. We selected HCCC-9810 cell lines for knockdown and RBE cell lines for overexpression of METTL3 due to their mRNA (Fig. 2A) and protein (Fig. 2B) expression patterns. Next, the efficacy of the two siRNAs in knocking down METTL3 expression was confirmed by RT‒qPCR (Fig. 2C) and western blotting (Fig. 2D). This deletion strategy resulted in impaired proliferation and elevated TUNEL-positive apoptotic cells in HCCC-9810 cells (Fig. 2E, F). Transwell assays showed similar impairments in migration and invasion in HCCC-9810 cells as a consequence of METTL3 knockdown (Fig. 2G). To determine whether METTL3 is necessary for aerobic glycolysis for the energy supply of CCA, we subjected siMETTL3 HCCC-9810 cells and siNC controls to glucose uptake and lactate production assays. As expected, METTL3 knockdown reduced the glycolytic response in HCCC-9810 cells, as evidenced by the decreased glucose uptake and lactate production (Fig. 2H, I). Therefore, METTL3 knockdown inhibits glycolysis and the malignant phenotype of CCA cells in vitro.

**Fig. 3:**
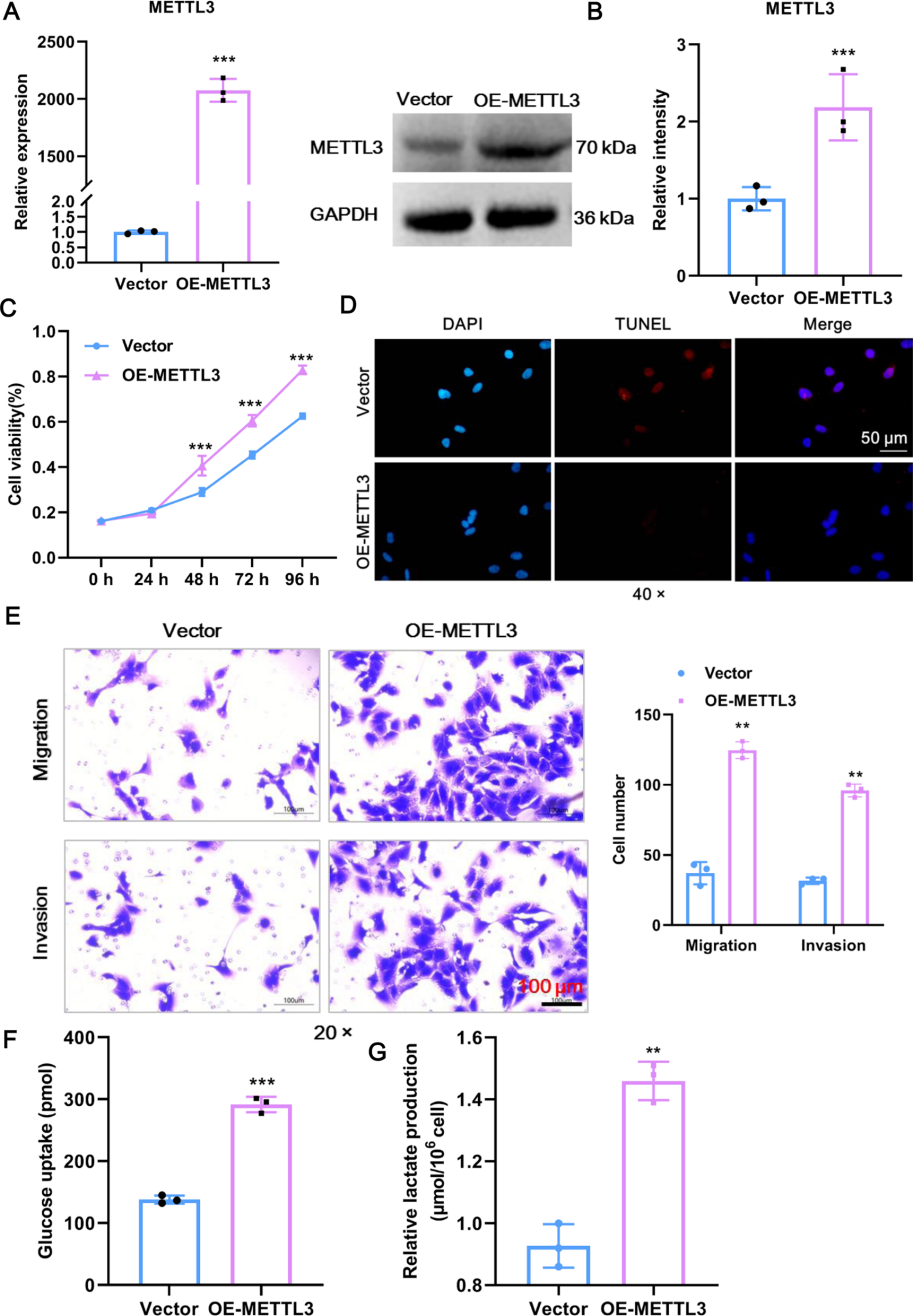
AKR1B10 promotes glycolysis and the malignant phenotype of CCA cells in vitro. **A** The overexpression efficacy of METTL3 mRNA expression in RBE cells was confirmed by RT‒qPCR. **B** The overexpression efficacy of METTL3 protein expression in RBE cells was confirmed by western blot. **C** The proliferation of RBE cells after overexpression of METTL3 was detected by CCK8. **D** TUNEL staining was used to detect RBE cell apoptosis after overexpression of METTL3. Scale bar: 50 μm. **E** The migration and invasion of RBE cells after overexpression of METTL3 were detected by Transwell assays. The numbers of migrating and invading cells were counted for comparison. Scale bar: 100 μm. **F** Glucose uptake of RBE cells after overexpression of METTL3 was detected using a commercial kit. (G) Lactate production in RBE cells after overexpression of METTL3. **Means P < 0.01, ***means P < 0.001

Following METTL3 knockdown in CCA cells, we next sought to determine the effects of METTL3 overexpression. For this, a lentivirus plus METTL3 plasmid (OE-METTL3) or empty vector controls (Vector) were transfected into RBE cells. The overexpression efficiency of METTL3 was confirmed at both the mRNA (Fig. 3A) and protein levels (Fig. 3B). The functionality of METTL3 overexpression produced precisely the opposite effect of METTL3 knockdown, as evidenced by the fact that cell proliferation, survival, migration, and invasion were boosted in the OE-METTL3 group relative to the Vector group (Fig. 3C–E). Moreover, high METTL3 expression was accompanied by spontaneous increases in glucose uptake and lactate production (Fig. 3F–G). Therefore, METTL3 overexpression promotes glycolysis and the malignant phenotype of CCA cells in vitro.

**Fig. 4:**
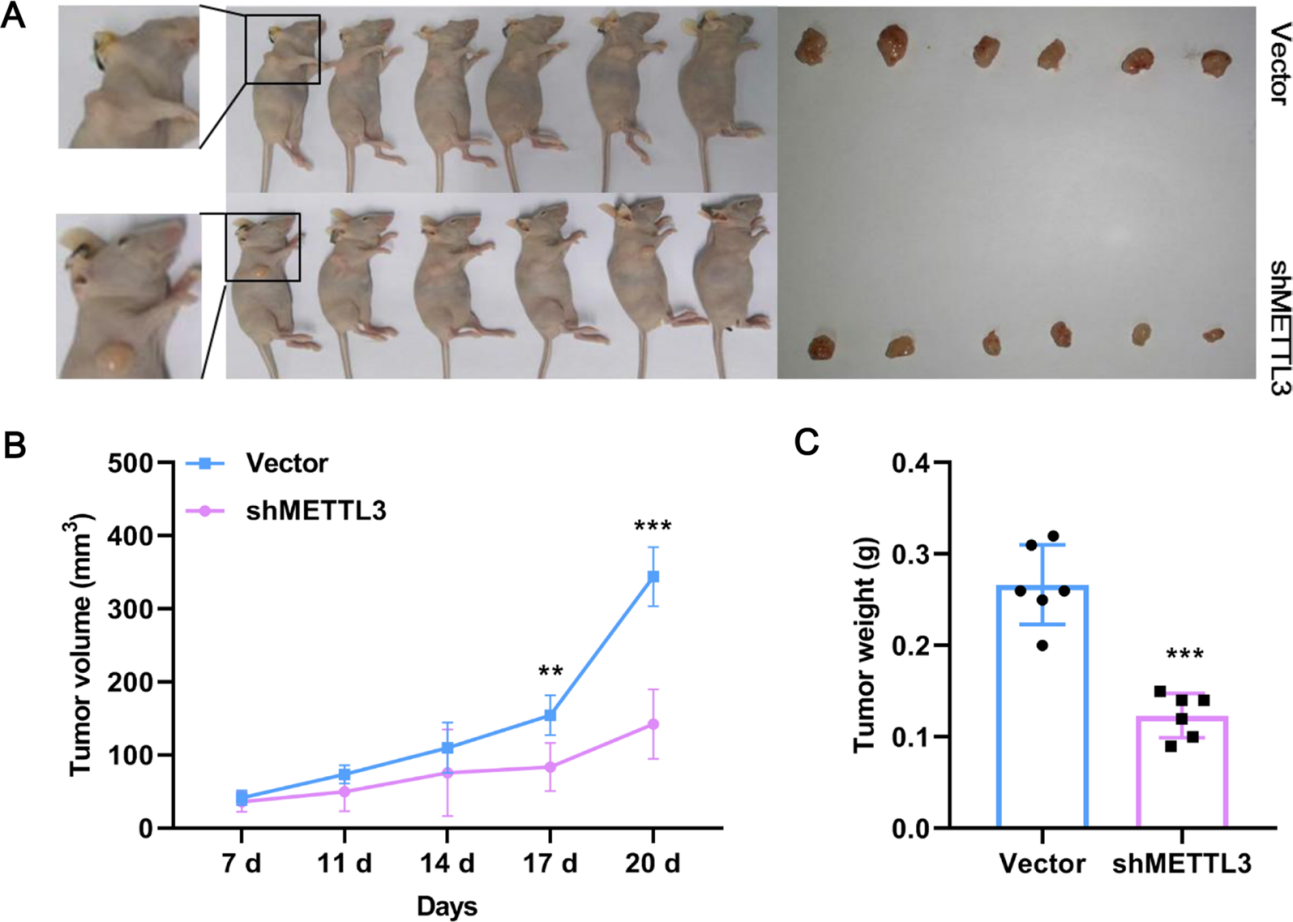
METTL3 promotes CCA tumor growth. **A** Representative image of mice. Knockdown of METTL3 effectively suppressed HCCC-9810 CCA cell subcutaneous tumor growth in nude mice (6 mice per group). **B** The relative tumor volume of mice (6 mice per group) was calculated every 3 days. **C** The tumor weight of mice (6 mice per group) was calculated. **Means P < 0.01, ***means P < 0.001

Demonstrating the promotion of CCA by METTL3 in vitro raised the question of whether METTL3 was also effective in vivo. For this, HCCC-9810 cells containing shMETTL3 or Vector were subcutaneously injected into the axilla of mice, and tumor growth was observed (Fig. 4A). Compared with Vector mice, shMETTL3 mice showed smaller tumor volumes and lighter tumor weights (Fig. 4B, C). Thus, METTL3 promotes CCA tumor growth in vivo.

### Whole-transcriptome sequencing reveals that AKR1B10 is a target of METTL3

**Fig. 5:**
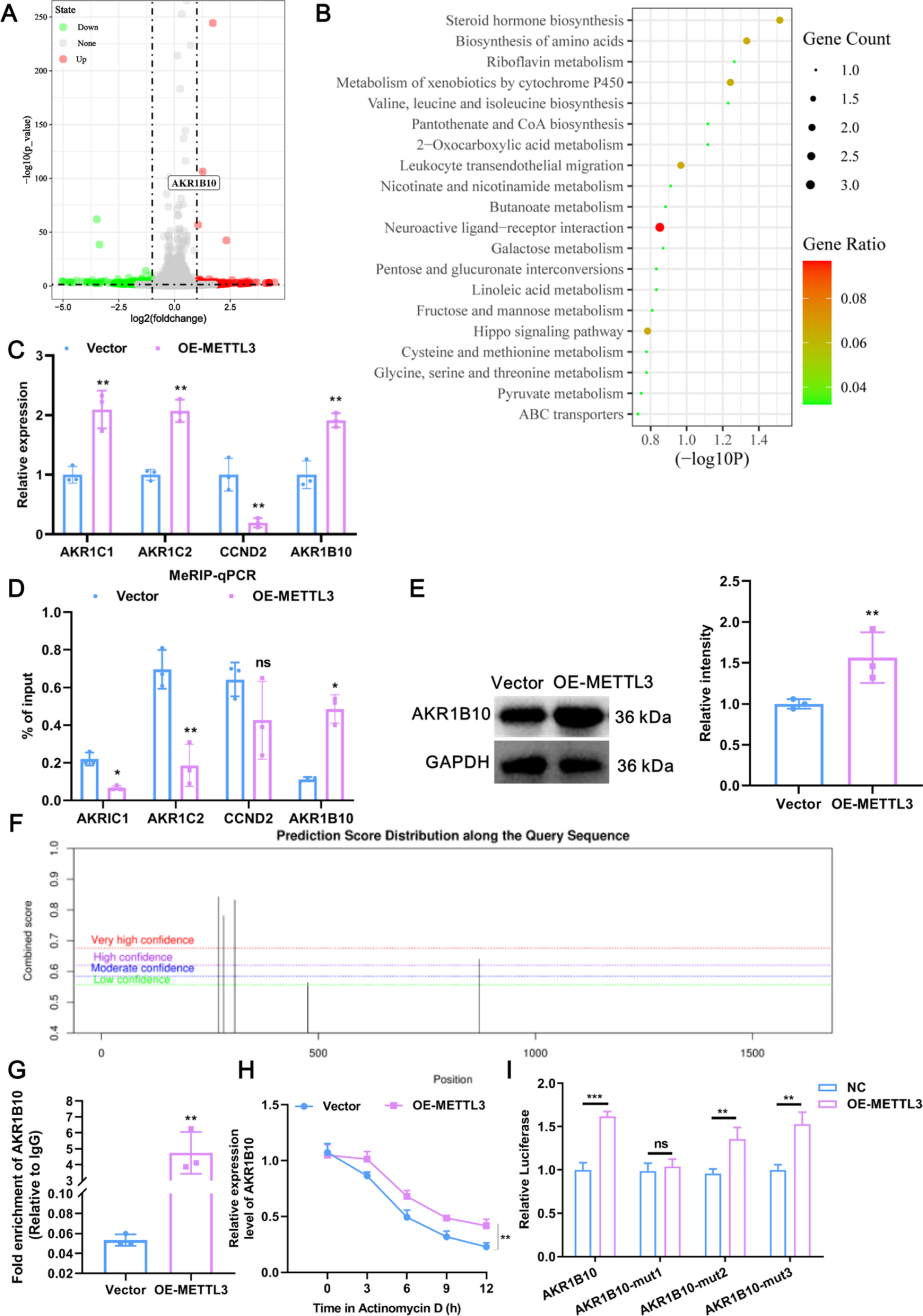
Whole-transcriptome sequencing reveals that AKR1B10 is a target of METTL3. **A** Volcano plots showing DEGs between METTL3-overexpressed and Vector groups by transcriptome sequencing in RBE cells (n = 3). Red dots means gene upregulated in METTL3-overexpressed group compared with the Vector group, such as AKR1B10; green dots means downregulated in METTL3-overexpressed group compared with the Vector group. **B** Bubble plot of KEGG enrichment of the upregulated DEGs between the METTL3-overexpressed and Vector groups in RBE cells. **C** RT‒qPCR verification of transcriptome sequencing results. Four upregulated DEGs between the METTL3-overexpressed and Vector groups were selected. **D** The results of MeRIP-qPCR in the OE-METTL3 and Vector groups for four candidate DEGs. **E** The protein expression of AKR1B10 in RBE cells upon METTL3 overexpression was detected by western blotting. **F** Five potential m6A modification sites on AKR1B10 mRNA predicted by the SRAMP database. **G** The binding relationship between METTL3 and AKR1B10 was detected by RIP assays. **H** The effect of METTL3 overexpression on the stability of AKR1B10 mRNA was measured by actinomycin D. **I** The m6A modification site of METTL3 on AKR1B10 mRNA was explored by using a luciferase reporter assay. ns means P > 0.05, *Means P < 0.05, **means P < 0.01, ***means P < 0.001

Next, to explore whether METTL3 can drive a spontaneous molecular alteration in CCA cells, we performed whole-transcriptome sequencing in RBE cells with or without METTL3 overexpression. Enforced expression of METTL3 resulted in changes in the expression profiles of 240 genes, of which 103 were upregulated, including AKR1B10, and 137 were downregulated (Fig. 5A). Interestingly, these DEGs were extensively involved in glycolytic metabolism-related pathways, including 2-oxocarboxylic acid metabolism, galactose metabolism, pyruvate metabolism, and carbon metabolism (Additional file 2: Fig. S1B). These results echo those of our previous conclusion that METTL3 promotes glycolysis. Given that METTL3 promotes the expression of downstream target genes through m6A modification [20], we focused on those DEGs that were upregulated upon METTL3 overexpression. All of the upregulated DEGs were subjected to KEGG analysis, and the results showed that the DEGs were primarily preferentially enriched in metabolic pathways, including galactose metabolism, butanoate metabolism, and riboflavin metabolism (Fig. 5B). To further determine the major affected molecules mediated by METTL3, we overlapped the genes upregulated in the METTL3-overexpression group from our whole-transcriptome sequencing and the genes upregulated in CCA tissues from TCGA, and then the intersection DEGs were analyzed by KEGG. The results showed that these intersection DEGs were mainly involved in glycolysis metabolism-related pathways, such as galactose metabolism, fructose and mannose metabolism, and glycerolipid metabolism (Additional file 2: Fig. S1C). Thus, 4 intersection DEGs (AKR1C1, AKR1C2, cyclin D2 (CCND2), and AKR1B10) related to energy metabolism were selected for RT‒qPCR validation. Except for CCND2, the remaining 3 DEGs were upregulated in the OE-METTL3 group compared to the Vector group (Fig. 5C). Moreover, MeRIP-qPCR results showed that a significant increase was observed only in AKR1B10 mRNA, which was precipitated by the m6A antibody, in the OE-METTL3 group compared with the Vector group, suggesting that AKR1B10 mRNA contains a METTL3-m6A modification site and that its m6A level was enhanced by METTL3 (Fig. 5D). Knockdown of METTL3 significantly reduced the m6A level of AKR1B10 (Additional file 2: Fig. S1D). The protein expression of AKR1B10 was also elevated upon METTL3 overexpression (Fig. 5E). Interestingly, according to SRAMP (http://www.cuilab.cn/sramp), there were indeed 5 potential m6A modification sites on AKR1B10 mRNA (Fig. 5F). Taken together, given our results above, we consider AKR1B10 to be a target of METTL3 and chose to further evaluate the role of AKR1B10 in CCA progression.

### METTL3 directly binds with AKR1B10 at an m6A modification site

To further confirm the binding relationship between METTL3 and AKR1B10, RIP assays were carried out. RIP results showed that AKR1B10 protein was specifically enriched by the METTL3 antibody (Fig. 5G). Ctinomycin D, a transcription inhibitor, was used to assess the stability of AKR1B10 mRNA upon METTL3 overexpression. We found that METTL3 overexpression significantly enhanced the stability of AKR1B10 mRNA compared with the control in the presence of actinomycin D (Fig. 5H). To explore the m6A modification site on AKR1B10, three AKR1B10 mutants with a single mutation were constructed. As shown in Fig. 5I, METTL3 significantly elevated the luciferase activity of the AKR1B10 wild-type luciferase reporter, as well as AKR1B10 mut-2 and mut-3, but not AKR1B10 mut-1. The results suggested that the binding between METTL3 and AKR1B10 was dependent on the m6A modification site of mut-1. Collectively, METTL3 promotes mRNA stability and increases the expression of AKR1B10 by directly binding in an m6A modification manner.

### AKR1B10 is highly expressed in CCA tissues

**Fig. 6:**
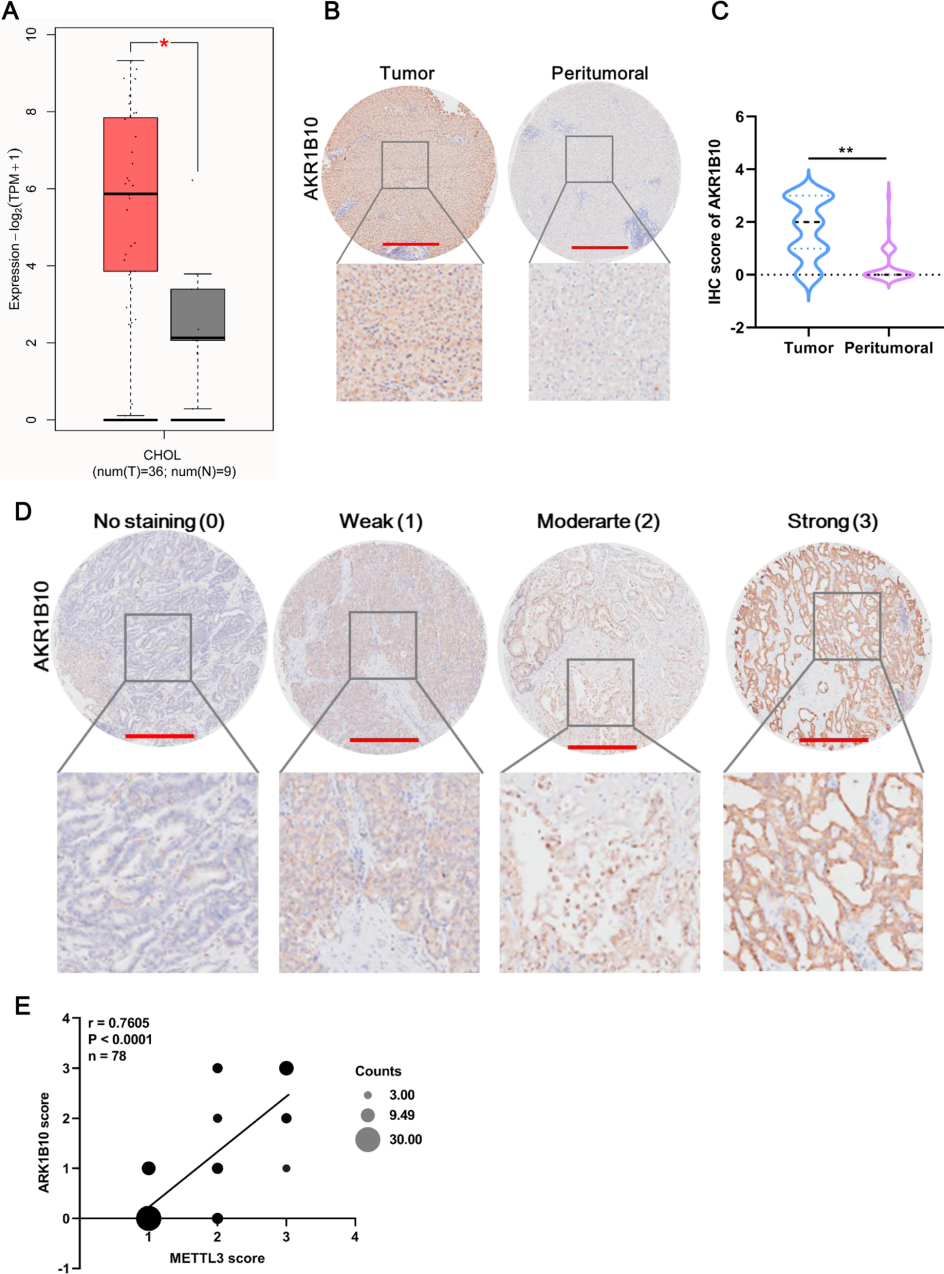
AKR1B10 is highly expressed in CCA tissues. **A** AKR1B10 expression in the GEPIA2 dataset. The red box indicates tumor tissue, and the gray box indicates normal tissue. **B** Representative image of AKR1B10 expression in tumor and peritumoral tissue in the CCA tissue microarray. Compared with that in peritumoral tissues, AKR1B10 was overexpressed in CCA tissues, as revealed by IHC staining for AKR1B10 in a CCA tissue microarray (n = 60). Scale bar: 500 μm. **C** The AKR1B10 score in CCA tissues was significantly higher than that in peritumoral tissues. The sample sizes were 60 in CCA tumor and peritumoral tissues. **D** Representative image of AKR1B10 expression at four levels in the CCA tissue microarray. AKR1B10 expression was scored at four levels (no staining, weak, moderate, strong) on the basis of IHC staining intensity. Scale bar: 500 μm. **E** Pearson correlation analysis of the IHC scores between METTL3 and AKR1B10. *Means P < 0.05, **means P < 0.01

We assessed AKR1B10 expression in CCA tissues using the GEPIA 2 database, and the results revealed that AKR1B10 was upregulated in CHOL tissue compared with normal tissue (Fig. 6A). A CHOL tissue microarray containing 60 samples also concluded that AKR1B10 was upregulated in CHOL tissues (Fig. 6B). Moreover, the overall IHC staining of AKR1B10 expression was scored according to 4 levels: no staining (0), weak (1), moderate (2), and strong (3) (Fig. 6D). Based on the IHC score, AKR1B10 expression in CHOL tissue was significantly higher than that in peritumoral tissue (Fig. 6C) and was not correlated with any clinicopathological characteristics (Table 1). In addition, IHC of the CHOL tissue microarray showed a highly significant positive correlation between the AKR1B10 score and the METTL3 score (Fig. 6E). Therefore, these results indicated that AKR1B10 is highly expressed in CCA.

### AKR1B10 promotes glycolysis and the malignant phenotype of CCA cells in vitro

**Fig. 7:**
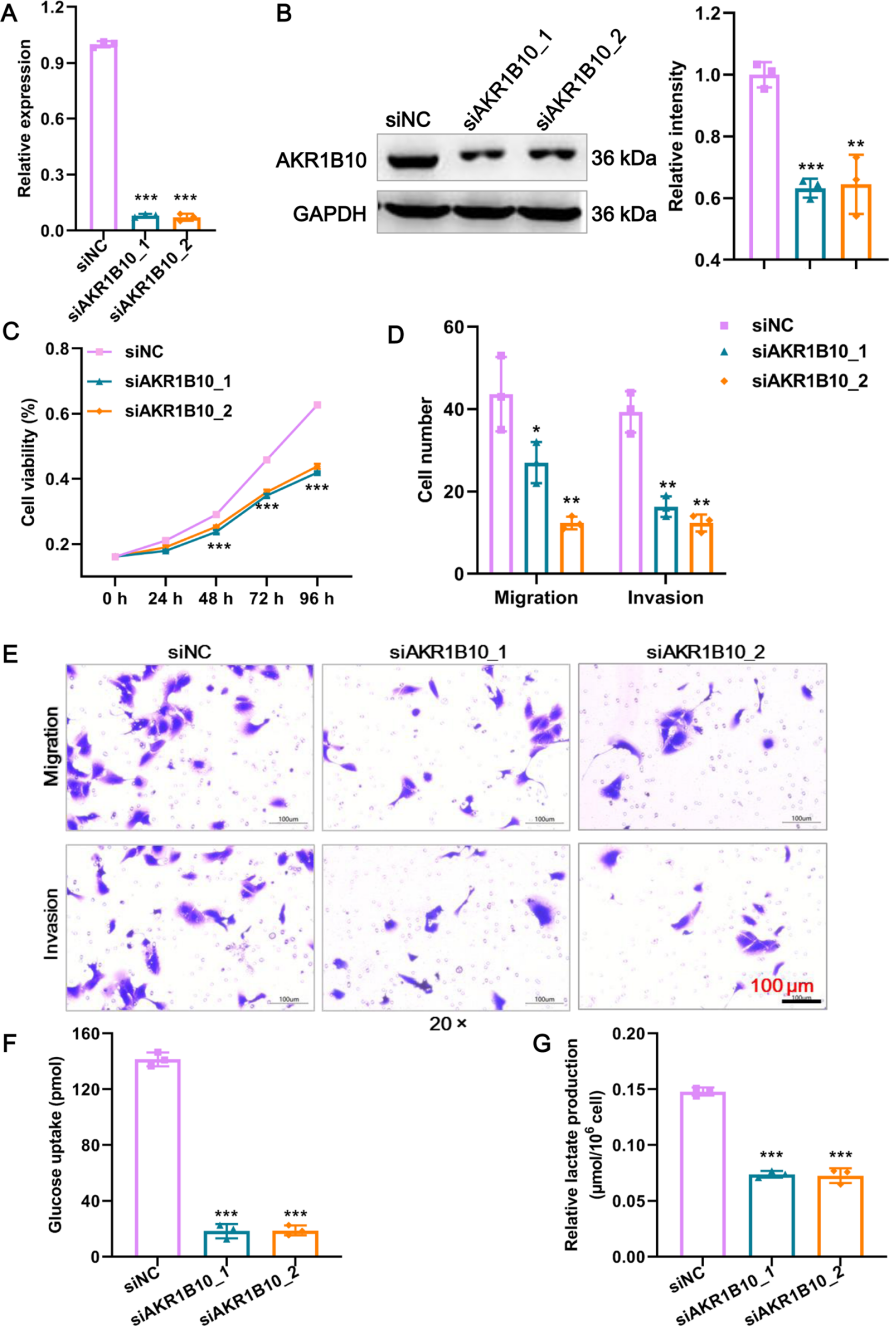
AKR1B10 promotes glycolysis and the malignant phenotype of CCA cells in vitro. **A** The knockdown efficacy of two siRNAs against AKR1B10 mRNA expression in RBE cells was confirmed by RT‒qPCR. **B** The knockdown efficacy of two siRNAs against AKR1B10 protein expression in RBE cells was confirmed by western blot. **C** The proliferation of RBE cells after AKR1B10 knockdown was detected by CCK8 assay. **D** Statistical results of Transwell assays. The numbers of migrating and invading cells were counted for comparison. **E** The migration and invasion of RBE cells after AKR1B10 knockdown were detected by Transwell assays. Scale bar: 100 μm. **F** Glucose uptake of RBE cells after AKR1B10 knockdown was detected using a commercial kit. **G** Lactate production in RBE cells after AKR1B10 knockdown. *Means P < 0.05, **means P < 0.01, ***means P < 0.001

To inquiry the function of AKR1B10 in CCA, we knocked down AKR1B10 expression in REB cell lines. The reduced expression of AKR1B10 in RBE cells was confirmed both at the mRNA (Fig. 7A) and protein levels (Fig. 7B). The CCK8 assay showed that siAKR1B10 led to impaired proliferation (Fig. 7C). Knockdown of AKR1B10 expression also resulted in significant declines in the invasive and migratory capabilities (Fig. 7D, E). Given that METTL3 regulates glycolysis and AKR1B10 mediates glycolysis, as reported in the literature [21], we examined the effect of AKR1B10 knockdown on glycolysis. As shown in Fig. 7F, G glucose uptake and lactate production were significantly reduced after AKR1B10 knockdown in RBE cells. In conclusion, AKR1B10 could promote glycolysis and the malignant phenotype of CCA cells in vitro.

### METTL3 exerts an oncogenic role in CCA through AKR1B10

**Fig. 8:**
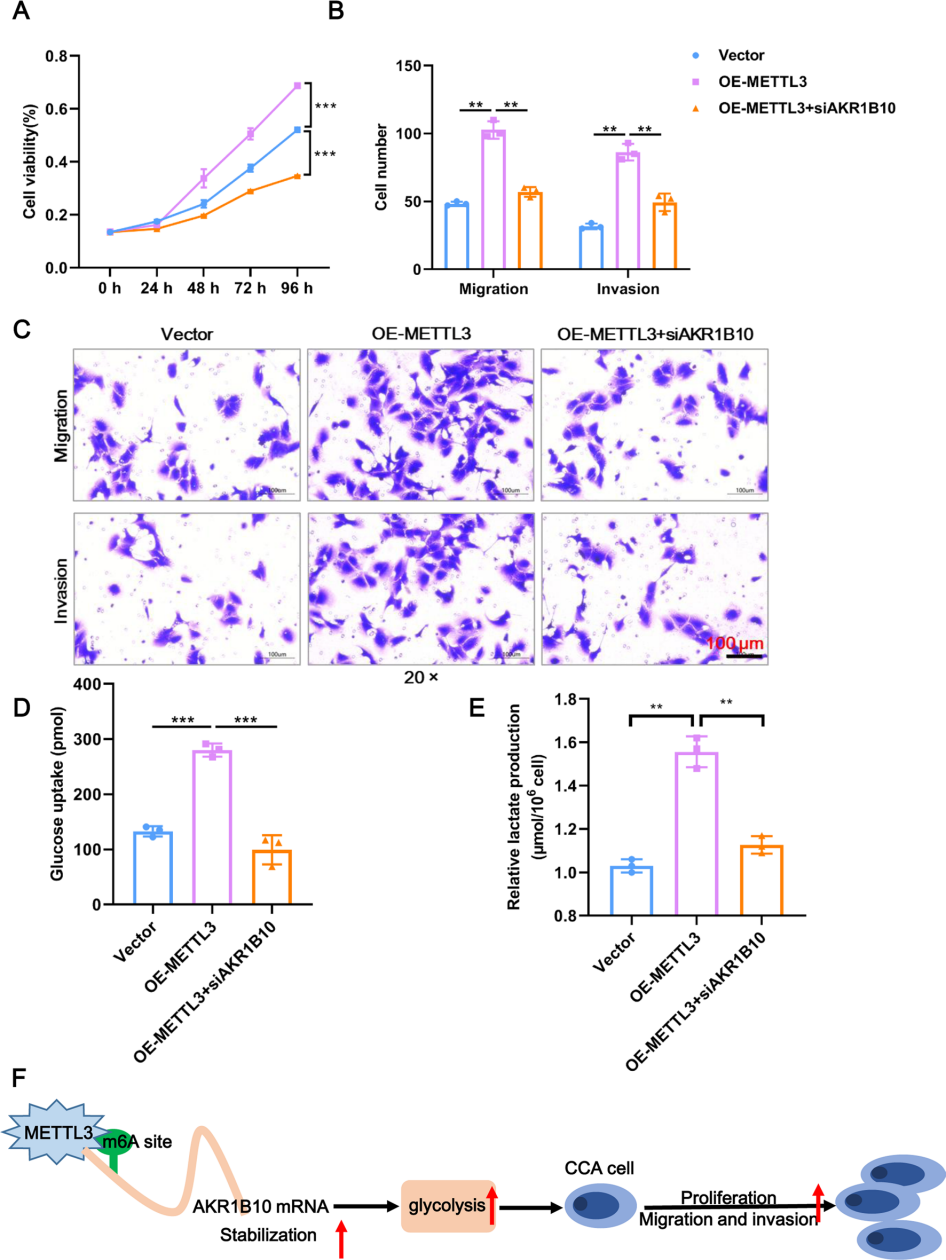
METTL3 exerts an oncogenic role in CCA through AKR1B10. **A** The proliferation of RBE cells after knockdown of AKR1B10 upon METTL3 overexpression was detected by CCK8 assay. **B** Statistical results of Transwell assays. The numbers of migrating and invading cells were counted for comparison. **C** The migration and invasion of RBE cells after knockdown of AKR1B10 upon METTL3 overexpression were detected by Transwell assays. Scale bar: 100 μm. **D** Glucose uptake of RBE cells after knockdown of AKR1B10 upon METTL3 overexpression was detected using a commercial kit. **E** Lactate production in RBE cells after knockdown of AKR1B10 upon METTL3 overexpression. *Means P < 0.05, **means P < 0.01, ***means P < 0.001

As confirmed in the preceding text, AKR1B10 is a target of METTL3, so we wondered whether METTL3 exerts an oncogenic role in CCA through AKR1B10. To this end, rescue assays of AKR1B10 silencing in METTL3-overexpressing cells were performed. As expected, METTL3 overexpression accelerated the proliferation, migration, and invasion in RBE cells compared to the Vector control group, but knockdown of AKR1B10 led to a partial reversal exhibited by the capacities for proliferation, migration, and invasion (Fig. 8A–C). Moreover, knockdown of AKR1B10 also reversed the glycolysis phenotypes of glucose uptake and lactate production caused by METTL3 overexpression (Fig. 8D–E). Therefore, METTL3 exerts an oncogenic role in CCA through AKR1B10.

## Discussion

CCA is a rare cancer but still affects a wide range of people, and its morbidity and mortality are increasing at alarming rates [22]. Numerous studies have revealed that m6A modification of RNA is tightly associated with the tumorigenesis and development of multiple cancers through various mechanisms, including CCA [23], bladder cancer [24], ovarian cancer [25], and liver cancer [26]. m6A methylation is catalyzed by a multicomponent methyltransferase complex that includes the m6A writer METTL3 [27]. In our study, we proved that METTL3 is highly expressed in CCA, METTL3 knockdown inhibits glycolysis and the malignant phenotype of CCA cells, and the same conclusion also holds for the METTL3 target gene AKR1B10. Moreover, AKR1B10 knockdown could rescue the effects of METTL3 overexpression on CCA cells. These data reveal that METTL3 promotes glycolysis and the malignant phenotype of CCA by mediating m6A modification of its target AKR1B10 (Fig. 8F).

METTL3 is the sole catalytic subunit in the m6A methyltransferase complex [28]. Depending on its m6A methyltransferase activity, METTL3 plays an essential role in tumor progression. For instance, METTL3 facilitated angiogenesis and carcinogenesis by m6A-mediated ADAMTS9 suppression in gastric cancer [29]. In colorectal cancer, METTL3 facilitated tumor metastasis by m6A-mediated methylation to enhance PLAU stability [30]. In bladder cancer, METTL3-mediated m6A modification regulates PD-L1 expression, resulting in resistance to CD8 + T-cell cytotoxicity and supporting tumor growth [31]. These results support the findings of our study. We found that METTL3 overexpression facilitated a malignant phenotype and glycolysis in CCA, and these functions were dependent on METTL3 m6A catalytic activity on AKR1B10. In addition, we retrieved only three references regarding the role of METTL3 in CCA. In the first, Ye et al. found that METTL3 and METTL14 combined with IGF2BP2 could promote CCA cell stemness by enhancing the stability and translation of CTNNB1 [23]. In the second, METTL3 facilitated intrahepatic CCA progression by accelerating IFIT2 decay in an YTHDF2-dependent manner [32]. In the third, 5-methylcytosine and METTL3-mediated m6A modification of lncRNA NKILA could accelerate the tumor growth and metastasis of CCA [33]. These results once again supported our conclusion. Overall, this work demonstrates for the first time that METTL3 facilitates the malignant phenotype of CCA by mediating m6A modification of AKR1B10 through the glycolytic pathway.

AKR1B10 is an aldo–keto reductase and is dependent on NAD(P)(H) to catalyze its target. Emerging studies have identified that AKR1B10 can reduce a large number of endogenous carbonyl compounds, including retinal, isoprenyl aldehydes, cytotoxic aldehydes, and decrease glucose reductase activity characteristics [34]. As a multifunctional reductase, AKR1B10 contributes to the maintenance of cellular homeostasis. An increasing number of studies have shown that AKR1B10 elevation is responsible for certain cancers. For example, AKR1B10 is significantly upregulated in cancers of the breast, lungs, and liver, and AKR1B10 overexpression facilitates the malignant phenotypes of these cancers [35–38]. In this study, we demonstrated that AKR1B10 exhibits a similar expression pattern and tumor-promoting effects in CCA. However, there are very few data on the role of AKR1B10 in CCA. Heringlake et al. reported a high expression pattern of AKR1B10 in CCA but did not research its function [39]. Gao et al. revealed an oncogenic role of AKR1C1 but not AKR1B10 in human CCA [40]. The present study is the first to uncover the expression pattern and oncogenic role of AKR1B10 in CCA. In addition, to the best of our knowledge, m6A-related AKR1B10 in cancer has not been reported; therefore, our study provides the first evidence that the tumor-promoting function of METTL3 is dependent on the m6A modification of AKR1B10.

In conclusion, our study revealed that METTL3 was highly expressed in CCA and that elevated METTL3 expression was associated with poor prognosis. METTL3 exerted an oncogenic role in CCA progression in vitro and in vivo, which was also the case for AKR1B10. Moreover, AKR1B10 was an m6A-related target of METTL3, and knockdown of AKR1B10 rescued the tumor-promoting effects induced by METTL3 overexpression. Therefore, METTL3 may function as a novel therapeutic target for CCA.

## Supplementary Information

**Additional file 1: Table S1.** The primers used in this study.

**Additional file 2: Figure S1.** **A** METTL3 expression in different cancers in the TIMER2 online database. **B** Bubble plot of KEGG enrichment of glycolysis-related pathways involved in DEGs. **C** Bubble plot of KEGG enrichment of intersection DEGs between the RNA-seq and TCGA datasets. **D** The results of MeRIP-qPCR in the siMETTL3 and siNC groups for four candidate DEGs.

## Data Availability

The datasets used and/or analyzed during the current study are available from the corresponding author on reasonable request.
