# Supplementary material for: METTL3 promotes glycolysis and cholangiocarcinoma progression by mediating the m6A modification of AKR1B10
Source: Cancer Cell Int. 2022 Dec 7;22:385. doi: 10.1186/s12935-022-02809-2 (PMC9730622; doi:10.1186/s12935-022-02809-2)
Supplement: Supplementary file 1 — Additional file 1: Table S1. The primers used in this study. [file 12935_2022_2809_MOESM1_ESM.docx]

Additional file 1: Table S1. The primers used in this study.

| Gene | Primers (5’ - 3’) | |
| --- | --- | --- |
| METTL3-F | TTTCCGGTTAGCCTTCGGGG | |
| METTL3-R | CATCCTAGTCTCCCAGCCCT | |
| Actin-F | AGCACAGAGCCTCGCCTTTG | |
| Actin-R | CTTCTGACCCATGCCCACCA | |
| AKR1B10-F | GTGACACCAGCACGCATTG | |
| AKR1B10-R | GCATTGAAGGGATAGTCTTCCAA | |
| AKR1C1-F | TCCAGTGTCTGTAAAGCCAGG | |
| AKR1C1-R | CCAGCAGTTTTCTCTGGTTGAA | |
| AKR1C2-F | TCCGTGATTGCATGTCTACAAGA |  |
| AKR1C2-R | GCAGGTTTTCTGGACACCAC |  |
| CCND2-F | TTTGCCATGTACCCACCGTC |  |
| CCND2-R | AGGGCATCACAAGTGAGCG |  |
| GAPDH-F | AGAAGGCTGGGGCTCATT | |
| GAPDH-R | TGCTAAGCAGTTGGTGGTG | |
| siNC | UUCUCCGAACGUGUCACGUTT | |
|  | ACGUGACACGUUCGGAGAATT | |
| siMEETL3 | GCAAGAAUUCUGUGACUAUTT | |
|  | AUAGUCACAGAAUUCUUGCTT | |
|  | GUUGAAAGGCAUUGAGAAUCU | |
| siAKR1B10 | UCAAUGUGCCGAUAUCCUGCA | |
|  | CAGGAUAUCGGCACAUUGACU | |

Note: F means forward primers, R means reverse primers.
